# The Inhibition of the Small-Conductance Ca^2+^-Activated Potassium Channels Decreases the Sinus Node Pacemaking during Beta-Adrenergic Activation

**DOI:** 10.3390/ph15030313

**Published:** 2022-03-04

**Authors:** Gergő Bitay, Noémi Tóth, Szilvia Déri, Jozefina Szlovák, Zsófia Kohajda, András Varró, Norbert Nagy

**Affiliations:** 1 Department of Pharmacology and Pharmacotherapy, Albert Szent-Györgyi Medical School, University of Szeged, 6720 Szeged, Hungary; geribitay@gmail.com (G.B.); toth.noemi@med.u-szeged.hu (N.T.); deri.szilvia@med.u-szeged.hu (S.D.); szlovak.j93@gmail.com (J.S.); varro.andras@med.u-szeged.hu (A.V.); 2 ELKH-SZTE Research Group of Cardiovascular Pharmacology, Hungarian Academy of Sciences, 6725 Szeged, Hungary; kohajda.zsofia@med.u-szeged.hu; 3 Department of Pharmacology and Pharmacotherapy, Interdisciplinary Excellence Centre, University of Szeged, 6720 Szeged, Hungary

**Keywords:** SAN pacemaking, small-conductance Ca^2+^-activated K^+^-channels, ISK, heart rate

## Abstract

Sinus pacemaking is based on tight cooperation of intracellular Ca^2+^ handling and surface membrane ion channels. An important player of this synergistic crosstalk could be the small-conductance Ca^2+^-activated K^+^-channel (I_SK_) that could contribute to the sinoatrial node (SAN) pacemaking driven by the intracellular Ca^2+^ changes under normal conditions and beta-adrenergic activation, however, the exact role is not fully clarified. SK2 channel expression was verified by immunoblot technique in rabbit SAN cells. Ionic currents and action potentials were measured by patch-clamp technique. The ECG R-R intervals were obtained by Langendorff-perfusion method on a rabbit heart. Apamin, a selective inhibitor of SK channels, was used during the experiments. Patch-clamp experiments revealed an apamin-sensitive current. When 100 nM apamin was applied, we found no change in the action potential nor in the ECG R-R interval. In experiments where isoproterenol was employed, apamin increased the cycle length of the SAN action potentials and enhanced the ECG R-R interval. Apamin did not amplify the cycle length variability or ECG R-R interval variability. Our data indicate that I_SK_ has no role under normal condition, however, it moderately contributes to the SAN automaticity under beta-adrenergic activation.

## 1. Introduction

The Ca^2+^-activated K^+^-current was first described in neurons in 1974 [1]. The existence and functional role of the Ca^2+^-activated K^+^-current in the heart was addressed in 1983 and it was concluded that there is no such current in the ventricular myocardium that carries exclusively K^+^-ions and is activated by intracellular Ca^2+^ [2]. In 2003, Xu et al. reiterated this issue and described a notable small-conductance Ca^2+^-activated K^+^-current (I_SK_) in ventricular and atrial myocytes of mice and human [3]. Recently, it was found that I_SK_ has an important role in several diseases and conditions such as atrial fibrillation, heart failure [4,5,6], cardiac memory [7,8], and J-wave syndrome [9]. Its important role was shown in normal atrial electrophysiology [10], however, no role was found in ventricular myocytes under normal conditions [11].

Considering that I_SK_ is a Ca^2+^-driven K^+^-current, theoretically it could have an important function in sinoatrial node (SAN) automaticity via providing a functional link between the intracellular Ca^2+^ and the repolarization process, especially under beta-adrenergic condition. However, the function of I_SK_ in SAN cells is poorly elucidated. Recently, Chen et al. and Torrente et al. demonstrated that I_SK_ contributes to the SAN pacemaking in normal rabbit and mouse SAN cells and NCX-knock out cell line [12,13]. In rabbit SAN cells, I_SK_ was even claimed as a key player of the pacemaking mechanism and action potential morphology.

The underlying mechanism of the SAN automaticity, after a long and intense debate, seems to be settled in a concept of the so-called coupled-clock mechanism [14]. It is widely accepted that SAN automaticity is based on the mutual crosstalk of the Ca^2+^-handling and surface membrane ion channels, where the ensemble of the membrane currents (I_CaL_, I_CaT_, I_Kr_, I_f_, I_NCX_) and the rhythmical Ca^2+^ releases from the sarcoplasmic reticulum (local Ca^2+^ releases, LCR, Ca^2+^-clock) form a robust, flexible coupled system, where neither part is dominant, and work synergistically [15]. The I_SK_, if functionally exists, could be a new component of this coupled-clock system.

Pharmacological control of the heart rate is a crucial intervention of the daily clinical practice in several diseases. However, the applied drugs, such as beta1-adrenoreceptor antagonists, funny current inhibitors (ivabradine), or L-type Ca^2+^ channels blockers (verapamil, diltiazem) have several limitations [16,17]. Since I_SK_ is a Ca^2+^-driven current, its contribution to pacemaking could be augmented under beta-adrenergic activation, but this issue was not previously examined. The aim of this study was to investigate the role of I_SK_ and its pharmacological inhibition under normal conditions as well as during beta-adrenergic activation in rabbit SAN cells and on isolated heart.

## 2. Results

### 2.1. Immunoblotting and Confocal Microscopy

**Figure 1:**
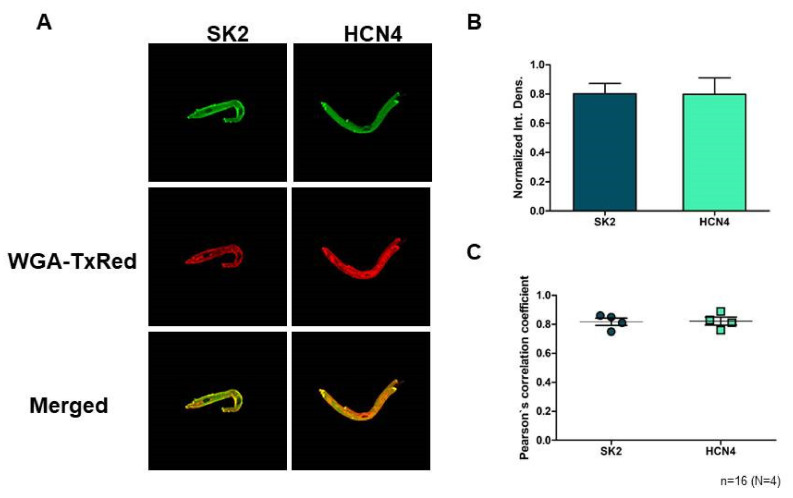
(**A**) shows representative immunofluorescent images of rabbit sinus node cells with SK2 and HCN4 immunolabelling. (**B**) shows the relative expression of SK2 and HCN4. There were no significant changes between the SK2 and HCN4 fluorescence intensity. (**C**) shows fluorescence colocalization analysis. One data point represents one animal. Four cells were evaluated and averaged from each animal. Data are presented as mean ± SEM, applied statistical probe was unpaired Student’s *t*-test (*p* ≤ 0.05).

SK2 expression was directly detected on isolated sinus node cells. Confocal microscopy on immunostained sinus node cells revealed abundant expression of SK2 channels on the surface membrane (Figure 1A). The SK2 fluorescence intensity does not show difference from the sinus node-specific HCN4 (*n* = 16 cells/4 animals; *p* = 0.97; Figure 1B). Colocalization of the SK2 immunofluorescence with WGA Texas-Red fluorescence was then analyzed by Pearson’s Correlation Coefficient (PCC) and we found that average PCC values of SK2 (0.82; (0.75–0.86)) were similar with HCN4 (0.82; (0.76–0.89); *n* = 16 cells/4 animals; *p* = 0.89; Figure 1C). These data suggest that SK2 is represented in the membrane of sinus node cells as HCN4.

### 2.2. Measurement of the Apamin-Sensitive Current

**Figure 2:**
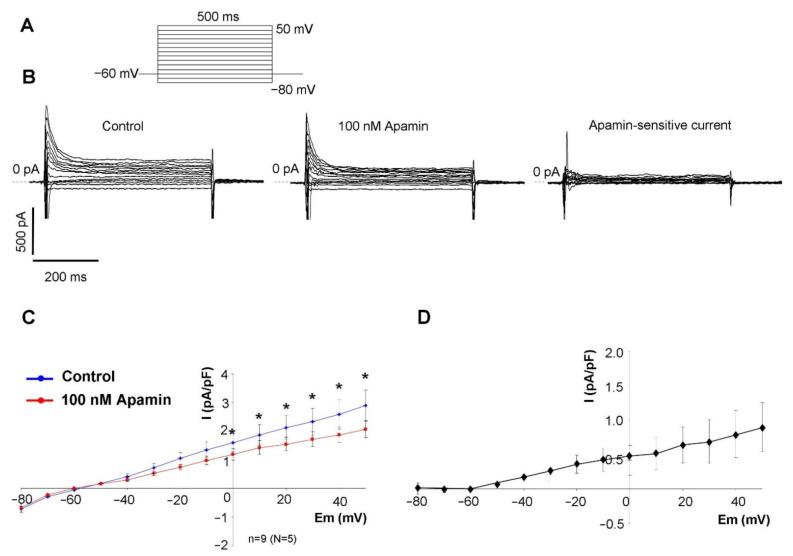
Characterization of the apamin-sensitive current. (**A**) illustrates the applied voltage protocol. (**B**) shows total membrane current under control condition (left curves), after 100 nM apamin application (middle curves), and the apamin sensitive current (right curves). (**C**) shows the current-voltage diagram of the control and apamin-treated currents and (**D**) illustrates the current-voltage relationship of the apamin-sensitive current. Statistical analysis was performed by paired *t*-test (*p* < 0.05).

In the first set of electrophysiological measurements, we aimed to investigate the potential existence of I_SK_ in SAN cells. In order to address this issue, a selective inhibitor apamin was employed in 100 nM concentration, which fully inhibits all three isoforms of the SK-channels. As SK-channels carry Ca^2+^-sensitive current, the intracellular Ca^2+^ level was set to 500 nM to activate the channels by using an appropriate mixture of EGTA and CaCl_2_. Rectangular voltage steps were applied from −80 mV to 50 mV from a holding potential of −60 mV. Application of apamin revealed a time-independent apamin-sensitive current within the membrane potential range of −40 to 50 mV (Figure 2).

**Figure 3:**
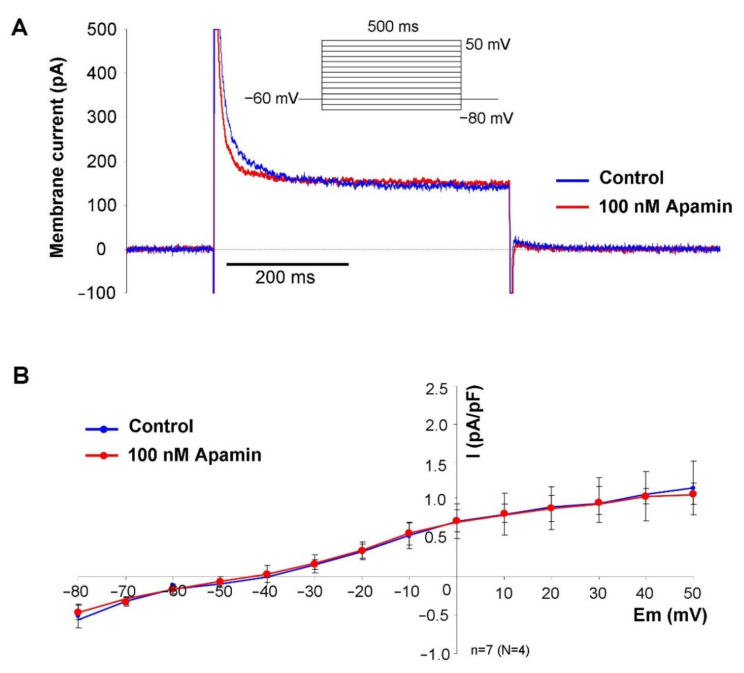
Characterization of the apamin-sensitive current. For further validation, the intracellular solution was heavily buffered with 10 mM BAPTA. Under this condition, the apamin failed to dissect any current from the control recording. (**A**) shows representative current traces under control condition (blue curve) and in the presence of 100 nM apamin (red curve) at 20 mV voltage pulse. (**B**) shows the current-voltage diagram where control and apamin resulted in identical curves. Statistical analysis was performed by paired *t*-test.

For further validation of the current, in the next set of experiments we buffered the intracellular Ca^2+^ with 10 mM BAPTA, and the same voltage protocol was applied (Figure 3). In this case, 100 nM apamin failed to dissect any current fragment from the control current, indicating that no I_SK_ was activated without free intracellular Ca^2+^.

**Figure 4:**
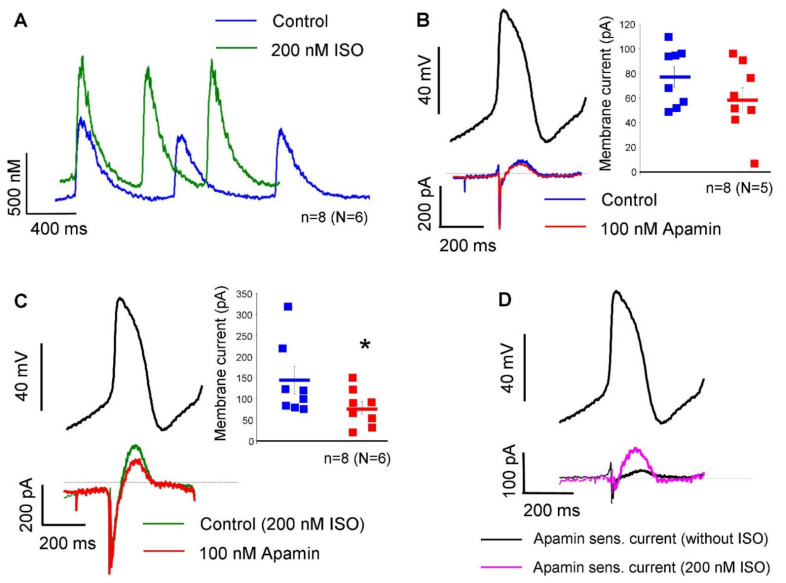
Measurement of apamin-sensitive current under a representative SAN action potential waveform in the absence and in the presence of 200 nM isoproterenol (ISO). (**A**) demonstrates the effect of 200 nM isoproterenol on Ca^2+^ transients. When isoproterenol was omitted, 100 nM apamin (red curve) did not alter the peak of the total current (blue curve, panel (**B**)). In the presence of 200 nM isoproterenol, 100 nM apamin (red curve) decreased the peak current (green curve, panel (**C**)). Representative original traces were determined after subtraction of currents. When 200 nM isoproterenol was employed, the apamin-sensitive current (purple curve) was larger than under normal conditions (black curve, panel (**D**)). Paired and unpaired *t*-tests (*p* < 0.05).

The apamin-sensitive current was also determined under a representative SAN action potential waveform, in the absence and in the presence of 200 nM isoproterenol without using Ca^2+^-chelators in the patch pipette. Figure 4A demonstrates that 200 nM isoproterenol did not change the diastolic Ca^2+^ level (104 ± 30 nM → 131 ± 33 nM; *n* = 8, N = 6; *p* = 0.13). The systolic peak was 566 ± 99 nM under control condition and was significantly increased to 732 ± 112 nM after isoproterenol application (*n* = 8, N = 6; *p* < 0.05). In the absence of isoproterenol, 100 nM apamin failed to significantly alter the peak of the total current (76 ± 8 pA → 59 ± 9 pA; *n* = 8, N = 5; panel B). In contrast, when 200 nM isoproterenol was applied previously, 100 nM apamin markedly decreased the peak of total current (144 ± 33 pA → 78 ± 15 pA; *p* < 0.05; *n* = 8, N = 6; panel C). Panel D demonstrates the comparison of apamin-sensitive currents in the absence (black curve) and in the presence of 200 nM isoproterenol (18 ± 6 pA vs 65 ± 20 pA; *p* < 0.05). The average cell capacitance of the current measurements was: 56.7 ± 4 pA/pF (*n* = 32).

Since the SK-channels are expected to carry a functional current depending on the intracellular Ca^2^, they may contribute to the SAN action potential and pacemaking in rabbit-isolated SAN cells. This assumption was investigated in the subsequent experiments.

### 2.3. The I_SK_ Has No Role in the SAN Pacemaking under Normal Condition

**Figure 5:**
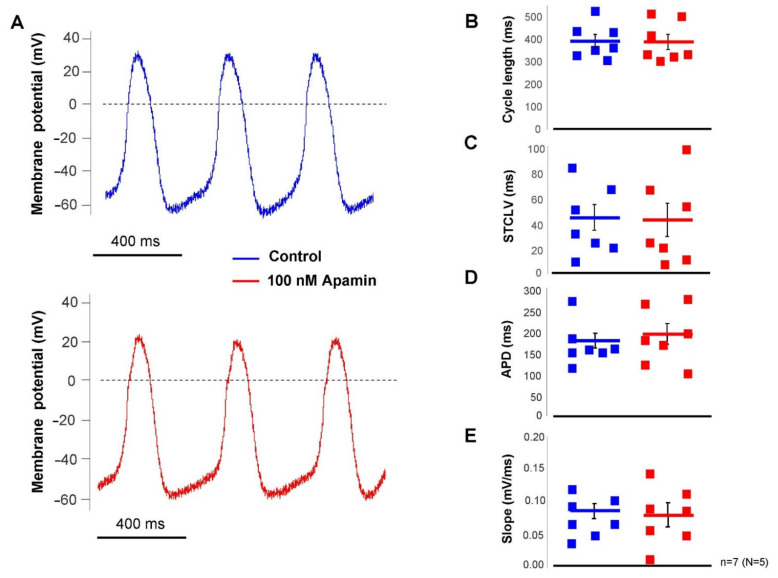
Action potential recordings from isolated SAN cells by perforated patch-clamp technique under normal condition. After control recordings (blue curve), 100 nM apamin was employed (red curve, (**A**). Bar graphs reported identical cycle lengths (**B**), short-term cycle length variability (**C**), APD (**D**), and slope of diastolic depolarization (**E**). Statistical analysis was performed by paired *t*-test.

The effect of selective SK-channel inhibition was tested in SAN pacemaking under basal condition (i.e., without beta-adrenergic modulation) by applying 100 nM apamin. Action potentials were measured by perforated patch-clamp technique from spontaneously beating SAN cells. It was found that apamin failed to influence any parameter of the SAN action potential (control → apamin; cycle length: 391 ± 30 → 388 ± 33 ms; cycle length variability: 43 ± 10 → 41 ± 13 ms; APD: 176 ± 17 → 193 ± 25 ms; slope of diastolic depolarization: 0.08 ± 0.01 → 0.08 ± 0.01 ms; *n* = 7, Figure 5).

### 2.4. I_SK_ Contributes to the SAN Action Potential under Beta-Adrenergic Activation

**Figure 6:**
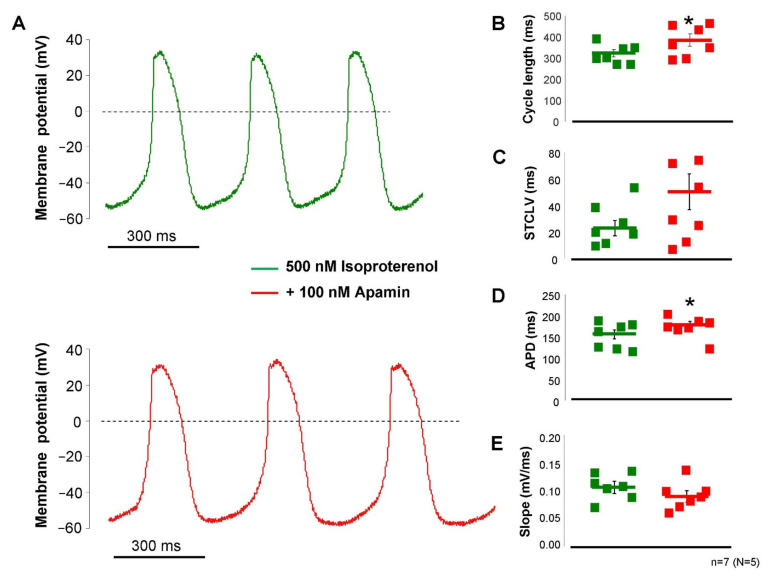
Action potential recordings from isolated SAN cells by perforated patch-clamp technique in the presence of 200 nM isoproterenol. The control recordings were measured in the presence of isoproterenol (green curve), then 100 nM apamin was employed (red curve, (**A**)). Application of apamin increased the cycle length (**B**) without alteration of the short-term cycle length variability (**C**), and increased the APD (**D**) without changing the diastolic depolarization (**E**). Statistical analysis was performed by paired *t*-test (*p* < 0.05).

The failure of apamin to influence SAN pacemaking demonstrated in Figure 5 indicated that the intracellular Ca^2+^ level may not reach an appropriate level to activate a sufficiently large current during the action potential. In this set of experiments, we activated the beta-receptors by the application of 200 nM isoproterenol to enhance the intracellular Ca^2+^ content of the cells. Under this condition (Figure 6), the application of 100 nM apamin significantly lengthened the cycle length (323 ± 17 → 387 ± 28 ms, *p* < 0.05, *n* = 7), without altering the short-term cycle length variability in a statistically significant manner (26 ± 6 → 40 ± 11 ms, *p* = 0.21, *n* = 7), but prolonged the APD (153 ± 10 → 176 ± 8 ms, *p* < 0.05, *n* = 7) and did not alter the slope of diastolic depolarization (0.11 ± 0.01 → 0.095 ± 0.01 mV/ms, *n* = 7). The maximal diastolic potential also did not show change upon apamin application (−56 ± 2 → 60 ± 3 mV, *n* = 7).

### 2.5. Inhibition of the I_SK_ Lengthens the ECG R-R Interval under Beta-Adrenergic Activation

**Figure 7:**
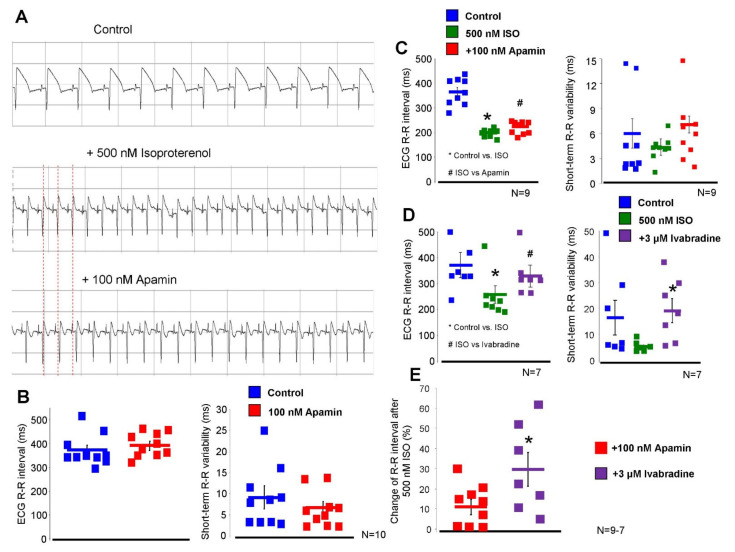
Results of Langendorff-perfused experiments on isolated hearts. (**A**) demonstrates original ECG traces in control condition (upper panel), in the presence of 200 nM isoproterenol (middle panel), and after apamin application (lower panel). Red vertical dashed lines indicate the change of the cycle lengths. (**B**) represents the basal condition (i.e., without application of isoproterenol). The ECG R-R interval (left graph) and the R-R variability (right graph) were unaltered after apamin administration. (**C**) represents apamin application in the presence of 500 nM isoproterenol. 100 nM apamin increased the ECG R-R interval (left graph), however, it did not change the R-R variability (right graph). (**D**) illustrates the effect of 3 µM ivabradine. Ivabradine markedly increases the R-R interval (left graph) and the R-R interval variability (right graph). (**E**) shows the comparison of the effects of apamin and ivabradine on the ECG R-R interval after 200 nM isoproterenol. Statistical analysis was performed by paired *t*-test and repeated measures ANOVA (*p* < 0.05).

The effect of 100 nM apamin was tested on *ex-vivo* isolated Langendorff-perfused hearts (Figure 7). During measurements, 40 consecutive ECG R-R intervals were measured and the mean R-R intervals and short-term R-R variability were calculated. Under normal conditions (i.e., without beta-adrenergic activation), 100 nM apamin failed to alter both the ECG R-R intervals (373 ± 20 → 393 ± 17 ms, *p* = 0.43, *n* = 10) and the R-R variability (6.4 ± 1.2 → 6.8 ± 1.4 ms, *n* = 10). Parallel with the apamin experiments, a time control group was measured where the vehicle of apamin was applied in order to detect any non-specific or time-dependent changes. No change was found in the ECG R-R interval (379 ± 10 → 373 ± 17 ms, *n* = 10) nor in the R-R variability (6.9 ± 1.6 → 7.1 ± 1.6 ms, *n* = 10).

In the second set of Langendorff experiments, we aimed to substantially activate the beta-adrenergic receptors in order to enhance the intracellular Ca^2+^ without causing arrhythmogenic events. Our preliminary experiments showed that 500 nM isoproterenol shortens the ECG R-R interval by 44 ± 4%, while arrhythmias were not observed. Under this condition, 100 nM apamin lengthened the ECG R-R interval (364 ± 17 → 200 ± 5 → 223 ± 10 ms, *p* < 0.05, *n* = 9) but did not alter the R-R variability (6.0 ± 1.7 → 4.4 ± 0.6 → 7.1 ± 3.8 ms, *p* = 0.41, *n* = 10). The parallel time control measurements exerted no statistically significant change in these variables upon administration of the vehicle (R-R interval: 370 ± 16 → 223 ± 8 → 231 ± 11 ms, *n* = 10; R-R variability: 8.1 ± 2.1 → 3.0 ± 0.3 → 4.0 ± 0.5 ms, *n* = 10).

In order to compare the magnitude and nature of the apamin effect to a well-known bradycardic agent, the effect of 3 µM ivabradine was examined in the presence of 500 nM isoproterenol. As it was expected, the application of ivabradine lengthened the ECG R-R interval (386 ± 48 → 257 ± 34 → 330 ± 41 ms, *p* < 0.05, *n* = 7), however, it also significantly increased the ECG R-R variability (11.7 ± 4.0 → 6.0 ± 0.7 → 20.0 ± 5 ms, *n* = 7).

## 3. Discussion

In this study we analyzed the possible role of the small-conductance Ca^2+^-activated K^+^-current under basal condition and during beta-adrenergic stimulation. Previous studies reported the functional role of SK-channels in SAN pacemaking without adrenergic stimulation. In contrast, in this study we failed to demonstrate a significant effect of SK-inhibition under normal condition, unless beta adrenergic stimulation was applied.

We do not know the discrepancy of the difference between our and the study of Chen at al. [12], but differences in the intracellular Ca^2+^ level cannot be ruled out between the two studies. Further investigations are necessary to resolve this issue.

The most important findings of this paper are: (i) the SK2-channels are expressed in rabbit SAN sarcolemma. (ii) The I_SK_ has no role in SAN pacemaking with basal intracellular Ca^2+^ level. (iii) Beta-adrenergic stimulation activates the I_SK_ and its inhibition causes moderate decrease in spontaneous automaticity via APD lengthening without increasing the cycle length variability.

### 3.1. The SK Channels Are Expressed in Rabbit SAN Myocytes

A previous paper demonstrated the expression of SK-channel in mice myocytes [13], however, based on our best knowledge this is the first data regarding the expression of SK2 in rabbit SAN cells, which is emphasized by the fact that the vast majority of SAN data are obtained from rabbit cells. Since a previous study indicated that the selective SK-channel inhibitor apamin exerts the highest sensitivity toward SK2 [18], the expression of SK2 isoform was elucidated in this study. Our experiments showed that the SK2 isoform is expressed in the surface membrane of rabbit SAN myocytes.

### 3.2. The Apamin-Sensitive Current Is Present in SAN Cells

SK-channels can be divided into three major subgroups: SK1 channels are encoded by KCNN1, SK2-channel encoded by KCNN2, and SK3 encoded by KCNN3 gene. Apamin, a polypeptide blocker of the I_SK_ isolated from bee venom, selectively blocks the current with isoform-dependent effectivity [19]. SK1 channels have the lowest sensitivity (IC_50_~10 nM); the SK3 is moderately sensitive (IC_50_~1 nM); and SK2 exerts the highest sensitivity (IC_50_~40 pM) [19]. This indicates that the applied 100 nM apamin in this study is far above the IC_50_-values of any channel subtypes, therefore, complete SK-channel block is expected in our experiments.

The SK-channels are suggested to be voltage-independent ion channels, activated by the rise of intracellular Ca^2+^ concentration, and carrying repolarizing K-currents [3]. Previous studies suggested that inward rectification of the SK-channels is caused by voltage-dependent block of intracellular divalent ions, however, a later study identified an intrinsic mechanism of channels causing inward rectification, which is independent of divalent ions [20].

Our patch clamp experiments revealed apamin-sensitive current in the voltage range between −40 to +40 mV when the intracellular Ca^2+^ was buffered to 500 nM. The half-maximal Ca^2+^ concentration for channel activation was reported as 300 nM [21], therefore, this Ca^2+^ concentration is suggested to cause nearly maximal current activation. When intracellular Ca^2+^ was buffered to 500 nM, the apamin-sensitive difference current was monotonously enhanced as the voltage was increased from −60 to + 40 mV. The current-voltage characteristic of the apamin-sensitive current both in terms of absolute current values and voltage-dependence was found to be similar to those that were illustrated in a previous study [13]. This current-voltage characteristic of the I_SK_ may imply that notable current could be expected above −20 mV, which corresponds to the main repolarization, at the same time it is plausible that marginal current is activated during the maximal diastolic potential. It is underpinned by the fact that modelling and experimental results indicate that intracellular Ca^2+^ level is the highest during early repolarization and considerable decays during the terminal phase of the repolarization [22,23]. Therefore, neither the membrane potential nor the intracellular Ca^2+^ favour I_SK_ activation at the maximal diastolic potential. In line with this, we did not find change in the maximal diastolic potential and any secondary change in the diastolic depolarization. As a negative control, highly buffered cells were used to demonstrate the Ca^2+^ sensitivity of the apamin-sensitive current. In contrast to high depolarization (i.e., +40 mV), apamin-sensitive current was not detected in the absence of free intracellular Ca^2+^.

For further validation, we analyzed the effect of apamin under a representative SAN action potential waveform without Ca^2+^ chelators in the patch pipette. In contrast to experiments with buffered Ca^2+^, we found negligible apamin-sensitive current under normal conditions in the presence of dynamic Ca^2+^ changes. Ca^2+^ transient measurements indicated that systolic peak of intracellular Ca^2+^ was barely larger than 500 nM and declined fast, which may induce small I_SK_. In contrast, when 200 nM isoproterenol was employed, intracellular Ca^2+^ was increased and apamin-sensitive current was considerably larger.

### 3.3. I_SK_ Has No Role in Sinus-Node Pacemaking under Normal Conditions but Its Inhibition Lengthens APD during Beta-Adrenergic Activation

Under normal conditions, apamin-induced AP alteration was not observed, which is in contrast to a previous report where ~18% cycle length increase was observed after apamin application under normal conditions in mouse SAN cells [13]. This extent of effect is comparable to the effect of I_f_ inhibition by ivabradine under normal conditions [24]. Similarly, apamin also failed to alter the ECG R-R interval in Langendorff-perfused hearts in our study without adrenergic stimulation. A recent clinical study also reported similar results. Forty-seven healthy male volunteers were examined during AP30663 (I_SK_ inhibitor) administration. AP30663 did not influence the R-R interval, regardless of the applied dose [25]. These results are in line with experiments demonstrating marginal apamin-sensitive current during the action potential in response to dynamic Ca^2+^ changes, under normal conditions.

Activation of the β-adrenoreceptors induces the adenylate-cyclase via stimulatory G-proteins, leading to increased intracellular cyclic AMP (cAMP) level. The elevated cAMP then increases the protein-kinase A level, which phosphorylates the L-type Ca^2+^ channels. The L-type Ca^2+^ channel-phosphorylation increases the Ca^2+^ influx, thus causing a net gain of the intracellular Ca^2+^. The elevated intracellular Ca^2+^ also increases the CaM/CaMKII axis, which is involved in several Ca^2+^-dependent processes [26,27,28]. Experiments demonstrated in Figure 4C,D suggest that the increased intracellular Ca^2+^ increases the current density of the apamin-sensitive current, where inhibition could influence the sinus-node pacemaking. In order to address this issue, the beta-receptor agonist, isoproterenol, was employed.

Application of apamin in the presence of beta-receptor activation caused a ~20% increase in the cycle length, while the slope of the diastolic depolarization was not changed. This result implies that the increase of cycle length after I_SK_ inhibition was attributable to the increased APD, but the diastolic depolarization was not changed. This effect could be attributable to: (i) the current-voltage diagram and apamin-sensitive current during the canonical action potential suggest marginal or no current in the voltage range of diastolic depolarization and (ii) the intracellular Ca^2+^ level is considerably decreased during the diastolic depolarization. In agreement with this result, the ECG R-R intervals of Langendorff-perfused hearts were increased by 12% after apamin administration when beta-receptors were activated.

### 3.4. I_SK_ inhibition Does Not Increase Cycle Length Variability

The rhythmicity of spontaneous action potential and ECG R-R interval was characterized by the short-term cycle length variability (in the case of action potentials) and short-term R-R variability (in the case of ECG measurements). The coupled-clock hypothesis claims that cycle length variability is an important indicator of the coupling between the Ca^2+^-clock and the surface membrane ion channels: When the synchronization between the two clocks is strong, the variability is low. Any intervention disrupting the Ca^2+^-clock and/or the membrane clock attenuates the coupling and leads to increased cycle length and cycle length variability [29]. In this study, neither the cellular measurements nor the isolated heart experiments show a significant increase in the cycle length variability after apamin administration. This result suggests that I_SK_ may not be involved in the ionic mechanism that directly governs the pacemaker mechanism. However, it may also imply that inhibition of I_SK_ under beta-adrenergic activation could be considered a safe intervention to decrease the heart rate.

The effect of apamin was compared to the effect of ivabradine, and it was found that the ECG R-R interval-increasing effect of apamin was 1/3 of the ivabradine (~12 vs. ~30%). This result implies that the significance of I_SK_ in controlling the heart rate is markedly smaller than the I_f_; this probably provides extra repolarizing current during beta-adrenergic stimulation, which may increase the flexibility of pacemaking. In contrast, ivabradine also significantly augmented the cycle length variability since I_f_ is a principal component of the coupled-clock mechanism.

## 4. Materials and Methods

### 4.1. Animals

New Zealand white rabbits from both genders weighing 2.0–2.5 kg were used for experiments obtained from a licensed supplier (Innovo Ltd., Budapest, Hungary).

### 4.2. Cell Isolation

Isolated single SAN cells were obtained by enzymatic dissociation. Rabbits were sacrificed by concussion after intravenous administration of 400 IU/kg heparin. The heart was rapidly removed and placed into a solution containing in mM: 135 NaCl, 4.7 KCl, 1.2 KH_2_PO_4_, 1.2 MgSO_4_, 10 HEPES, 4.4 NaHCO_3_, 10 glucose, and 1.8 CaCl_2_ (titrated to pH 7.2 with NaOH). The heart was mounted on a cc. 60 cm high modified Langendorff column and was perfused with oxygenated solution at 37 °C. In the initial period, the blood was washed out (3–5 min) from the heart, then it was continuously perfused with nominally Ca^2+^-free solution until the heart ceased contractions (cca. 10 min). The enzymatic dissociation was performed by using 1.8 mg/mL (260 U/mL) collagenase (type II, Worthington) and 33 µM CaCl_2_ in the perfusate. After 13–14 min, the heart was taken off the cannula. The right atrium of the heart was cut and the SAN region was excised and cut into small pieces. The strips were placed into enzyme free isolation solution containing 1 mM CaCl_2_ and equilibrated at 37 °C for 15 min. The cells were separated by filtering through a mesh and were stored at room temperature.

### 4.3. SK2 Immunocytochemistry

Rabbit sinus node cells were isolated from the sinus-node, then were fixed on glass coverslips with acetone. The cell membrane was labelled Wheat Germ Agglutinin Texas Red-X Conjugate (WGA-TxRed) (ThermoFischer, Waltham, MA, USA; 1:500). After membrane labelling, the samples were blocked for 1 h with PBST (PBS with 0.01% Tween) containing 2.5% BSA at room temperature. SK2 and HCN4 were labelled with anti-KCa2.2 (SK2) (Alomone, Jerusalem, Israel; 1:50) and anti-HCN4 (Alomone; 1:50) primary antibody overnight at 4 °C. Next day, the cells were incubated with Goat anti-Guinea Pig IgG Alexa Fluor 488 secondary antibody (ThermoFischer; 1:500) for 1 h at room temperature. Fluorescent images were captured by an LSM 880 (Zeiss, Oberkochen, Germany) laser scanning confocal microscope. Images were quantitatively analyzed by ImageJ software. Control samples were incubated with secondary antibody only (No Primary Control).

### 4.4. Measurement of Apamin-Sensitive Current

The isolated SAN cells were placed in a low volume chamber (RC47FSLP, Warner Instruments, Hamden, CT, USA). Five minutes were allowed for the cells to settle and adhere, and the solution was continuously superfused by a peristaltic pump (C.P.78012-45, Ismatec, Zurich, Switzerland). Patch-pipettes were created from borosilicate glass capillaries using a P-97 microelectrode puller (Sutter Instruments, Novato, CA, USA), having a tip resistance between 1.5–2.5 MΩ. Ionic currents were recorded by using Axopatch 200B amplifier (Molecular Devices, Sunnyvale, CA, USA). Membrane currents were digitalized by Digidata 1550B (Molecular Devices, Sunnyvale, CA, USA) with 250 kHz under software control (pClamp 10.0, Molecular Devices, Sunnyvale, CA, USA). The temperature of the measurements was kept at 37 °C by using TC-344B temperature controller (Warner Instruments, USA).

Apamin-sensitive current was measured by rectangular voltage pulses, or by representative SAN action potential waveform. Rectangular voltage steps before and after 100 nM apamin application were employed from a holding potential of −60 mV. The membrane was depolarized to 50 mV for 500 ms by using 10 mV voltage steps. The internal solution contained (in mM): KCl 40, K_2_ATP 5, HEPES 10, MgCl_2_ 5, and GTP 0.1 and was titrated to pH 7.2 by using KOH. The free Ca^2+^ concentration in the pipette solution was set to 500 nM by using an appropriate mixture of EGTA and CaCl_2_ (calculated by the Maxchelator software). In experiments where the intracellular Ca^2+^ was highly buffered, we used 10 mM BAPTA without applying CaCl_2_ in the pipette. The composition of the external solution was (in mM): NaCl 144, NaH_2_PO_4_ 0.4, KCl 4, MgSO_4_ 0.53, CaCl_2_ 1.8, HEPES 5, glucose 5.5, titrated to pH 7.4 with NaOH.

In another set of experiments, the cells were paced using a canonical AP waveform by the average of 10 independent action potentials from previously recorded perforated patch clamp experiments. The parameters of this representative AP waveform were: cycle length: 410 ms, AP duration: 180 ms, maximal diastolic potential: −57 mV, overshoot: 24 mV, and diastolic depolarization slope: 0.124 mV/ms. Two experimental groups were established. In the first group, the current was recorded first in normal Tyrode’s solution (as previously described) as the control current, then 100 nM apamin was applied. In the other experimental group, the solutions were supplemented with 200 nM isoproterenol to enhance the intracellular Ca^2+^ of the SAN cells. Apamin-sensitive current was determined as a difference current. The pipette solution was as previously described without chelators.

### 4.5. Measurement of Ca^2+^ Transients

Ca^2+^ transients were measured from spontaneously active isolated SAN cells. Cells were loaded with Fluo-4 AM (5 µM) fluorescent dye. The isolated cells were kept in darkness at room temperature and were loaded with the dye for 20 min. Fluorescence measurements were performed on the stage of an Olympus IX 71 inverted fluorescence microscope. The dye was excited at 480 nm and the emitted fluorescence was detected at 535 nm. Optical signals were sampled at 1 kHz and recorded by a photon counting photomultiplier (Hamamatsu, model H7828; Hamamatsu Photonics Deutschland GmbH, Herrsching am Ammersee, Germany). Optical signal was converted to voltage using photon-voltage converter (Ionoptix, Westwood, MA, USA) and analyzed by pClamp 10.0 (Molecular Devices, Sunnyvale, CA, USA). Spontaneously and rhythmically contracting cells were chosen, therefore, no external pacing was needed to record Ca^2+^ transients. Amplitudes of the measured Ca^2+^ transients were calculated as differences between systolic and diastolic values. To obtain maximal fluorescence (Fmax), the cells were damaged by a patch pipette at the end of the experiment. Ca^2+^ was calibrated using the following formula: Kd(F-Fmin)/(Fmax-F). The Kd of the Fluo-4 AM was set to 335 nM. The extracellular solution was normal Tyrode’s solution as was previously described.

### 4.6. Action Potential Measurements from Single Cells by Current Clamp Configuration

Action potentials were measured from spontaneously beating sinus-node cells by using the perforated patch-clamp technique as was described in a previous study [30]. Normal Tyrode’s solution was applied, containing (in mM): 144 NaCl, 0.4 NaH_2_PO_4_, 4 KCl, 0.53 MgSO_4_, 1.8 CaCl_2_, 5.5 glucose, and 5 HEPES, titrated to pH 7.4 with NaOH. The microelectrodes were filled with a solution that contained (in mM): 120 K-gluconate, 2.5 NaCl, 2.5 MgATP, 2.5 Na_2_ATP, 5 HEPES, 20 KCl, titrated to pH 7.2 with KOH. 35 µM β-escin was employed in the pipette solution to achieve the membrane patch perforation.

The parameters of the APs were calculated as follows:

(a) Maximum diastolic potential (MDP) was defined as the most negative potential before the next AP depolarization.
(b) Take off potential (TOP) was calculated as the voltage measured at the time when the voltage derivative exceeded 0.5 mV/ms.
(c) The slope of diastolic depolarization was defined as the mean voltage derivative of the AP between MDP and take off potential.
(d) Action potential duration (APD) was obtained as the time interval between TOP and the next MDP.
(e) Cycle length was calculated between the peaks of two consecutive APs.
(f) All experiments in this study were carried out at 37 °C.

### 4.7. Langendorff-Perfusion Measurements on Isolated Hearts

ECG of isolated rabbit hearts were obtained in Langendorff-perfused hearts as described before [31]. Rabbits were sacrificed by concussion after 400 IU heparin were injected intravenously. The excised hearts were mounted by the aorta on a Langendorff-apparatus and retrogradely perfused with modified Krebs-Henseleit bicarbonate buffer (KHB) at a constant pressure (80 Hgmm). The KHB solution contained (in mM): NaHCO3 25; KCl 4.3; NaCl 118.5; MgSO4 1.2; KH2PO4 1.2; glucose 10; CaCl2 1.8, having a pH of 7.4 ± 0.05 when gassed with 95% O_2_ + 5% CO_2_.

The electrical activity as electrocardiogram (ECG) was obtained by the three lead custom-made electrodes and signal amplifier (Experimetria Ltd., Budapest, Hungary). Signal processing and analysis was carried out using HaemoSys (Experimetria Ltd., Budapest, Hungary).

### 4.8. Statistics

Normal distribution of the data was verified by using Shapiro–Wilk test. Statistical significance (*p* < 0.05) was assessed using Student’s *t*-test and repeated measures ANOVA depending on the experiment. Data are presented as means ±S.E.M.

## 5. Conclusions

Our results indicate that I_SK_ has no or very limited role in SAN pacemaking under basal condition, however, it may contribute to the repolarization under beta-adrenergic activation. Selective inhibition of I_SK_ induces a moderate cycle length increase, with the latter effect being attributed to the lengthening of APD. Further in-vitro and in-vivo studies are required to estimate the possible therapeutic potential of I_SK_ inhibition as a supportive heart-rate-reducing agent.

## Data Availability

Data is contained within the article.
